# Supplementary material for: Thermal engineering of FAPbI3 perovskite material via radiative thermal annealing and in situ XRD
Source: Nat Commun. 2017 Jan 17;8:14075. doi: 10.1038/ncomms14075 (PMC5247577; doi:10.1038/ncomms14075)
Supplement: Supplementary Information — Supplementary Figures [file ncomms14075-s1.pdf]

Supplementary Information

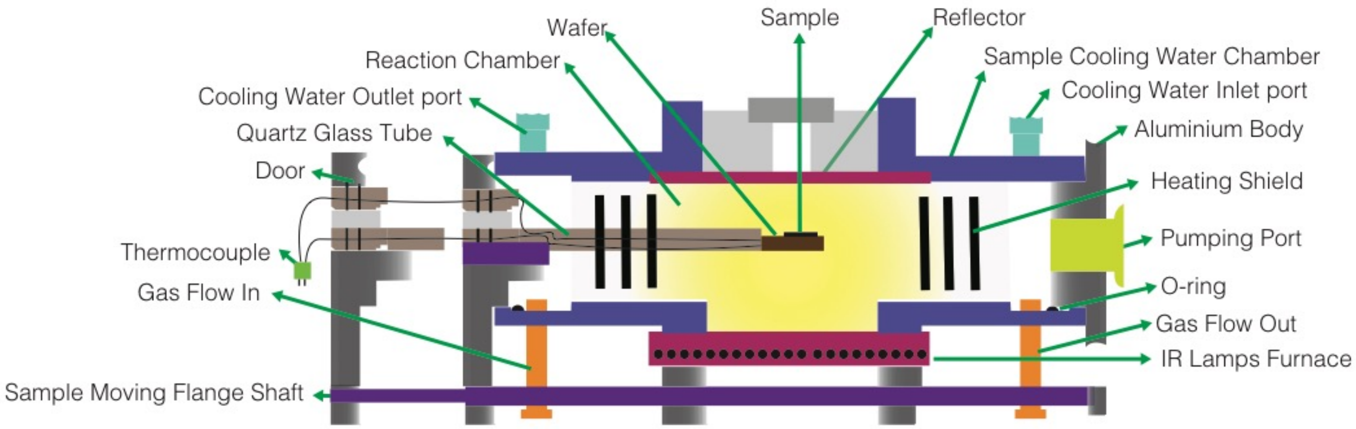

Supplementary Figure 1| Cross section of the RTA chamber used in the study to fabricate $FAPbI_3$ film and device.

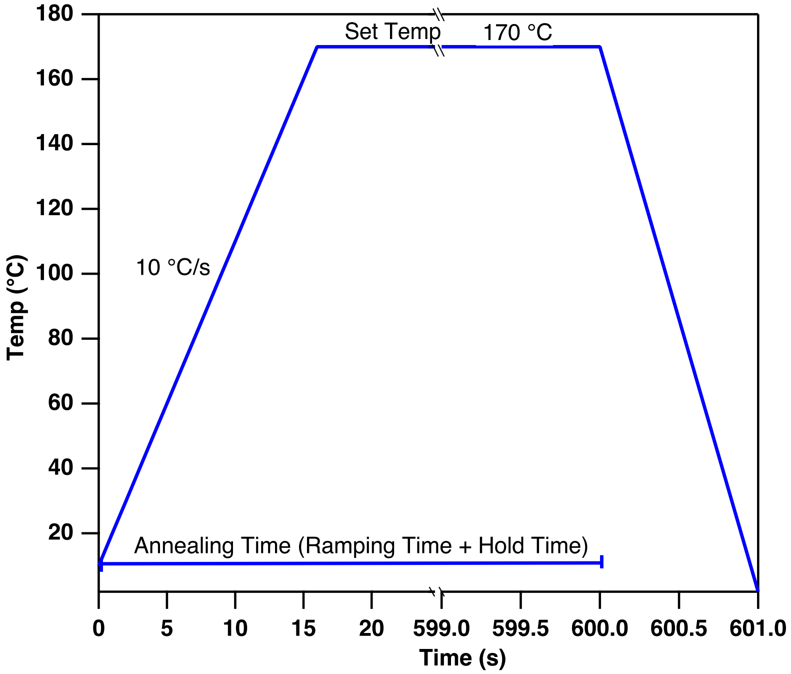

Supplementary Figure 2 | Temperature profile of the RTA devices with $FAPbI_3$ films fabricated at 170°C for 10 min.

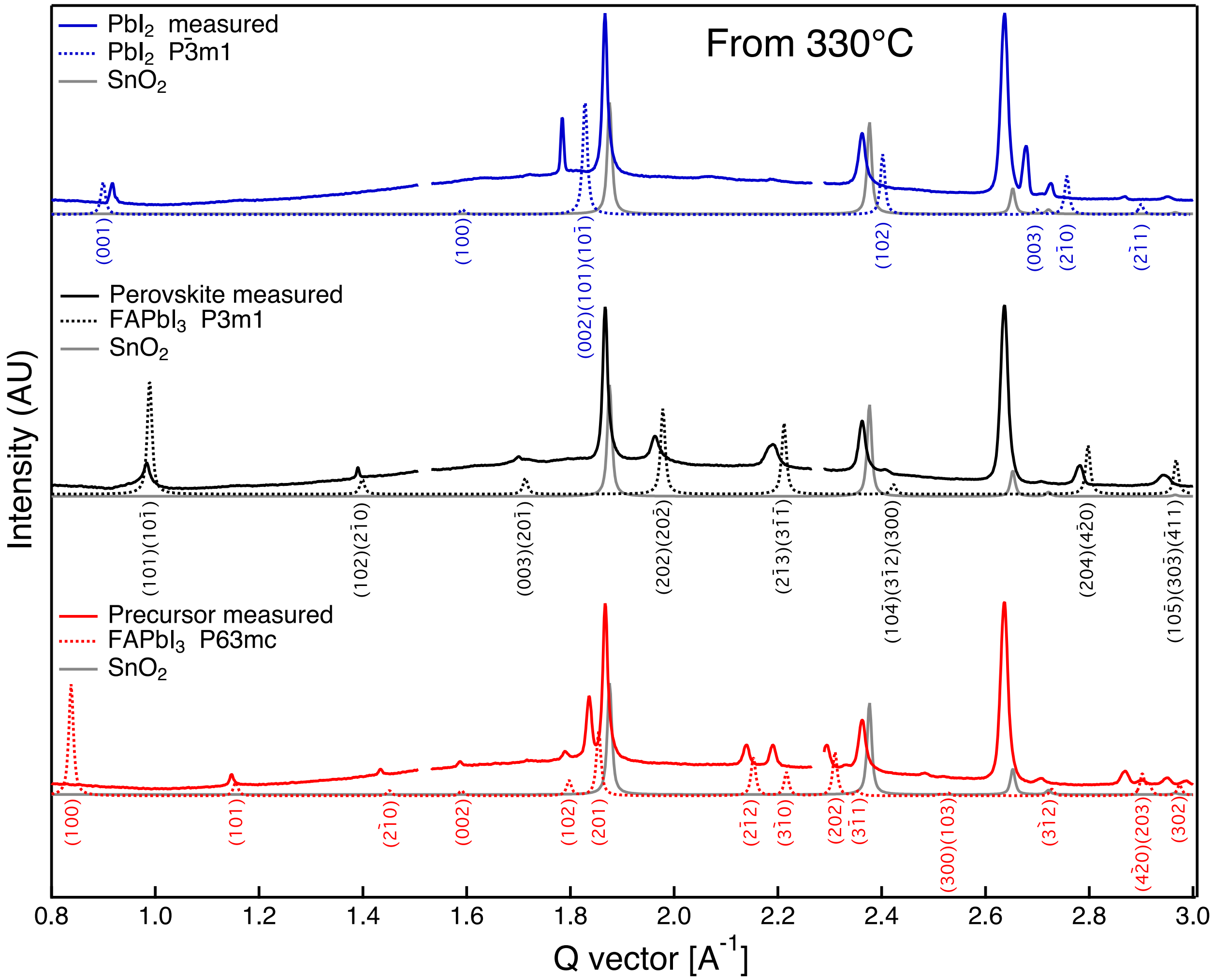

Supplementary Figure 3 | XRD full peak identification. From top to bottom, the X-ray data were obtained from 330 °C scan at 10.7 s when sample temperature is 117°C (blue solid line); 16.1 s when sample temperature is 171°C (black solid line) and 42.9 s when the temperature is 330 °C (red solid line). The standards shown are for room temperature, and consequently, the standard peak positions are at larger *Q* than the data due to thermal expansion in sample data.

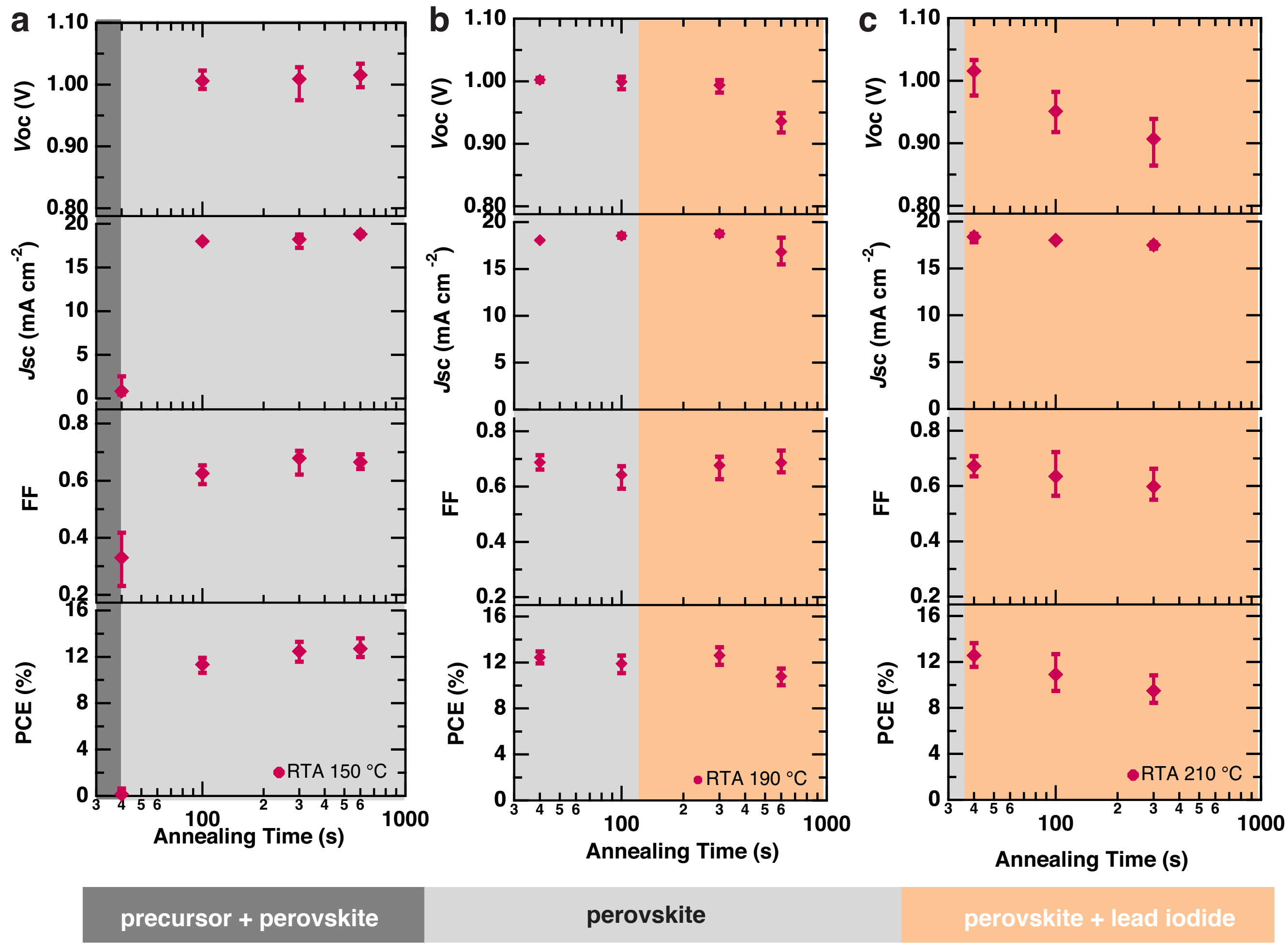

Supplementary Figure 4 | Photovoltaic performance of devices with active layer annealed at various temperatures and times. a, 150°C. b,190°C. c, 210°C. The statistics include average, maximum and minimum values, show by square dots and error bars. The different colors represent different phase stages as indicated in the legend. The device performance is from the reversely scanned *J-V* curves.

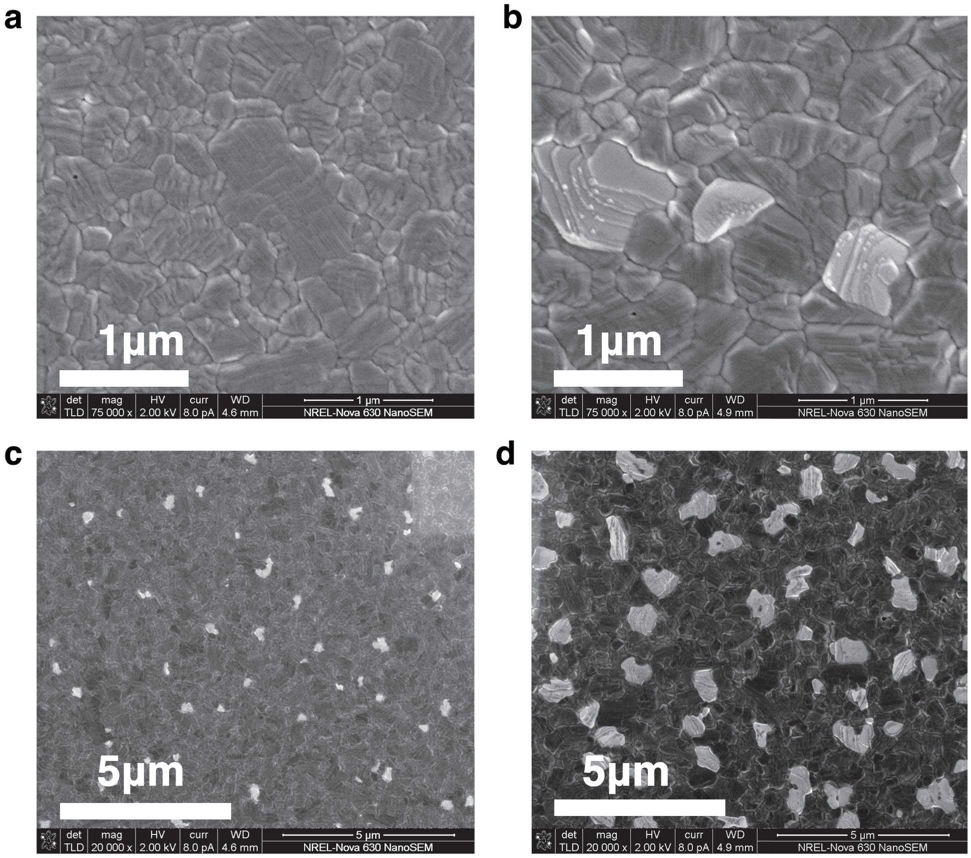

Supplementary Figure 5 | SEM of $FAPbI_3$ films annealing at 210 ° for different times. a, 40 s (scale bar: 1 μm). b, 5 min (scale bar: 1 μm). c, 40 s (scale bar: 5 μm). d, 5 min (scale bar: 5 μm.)

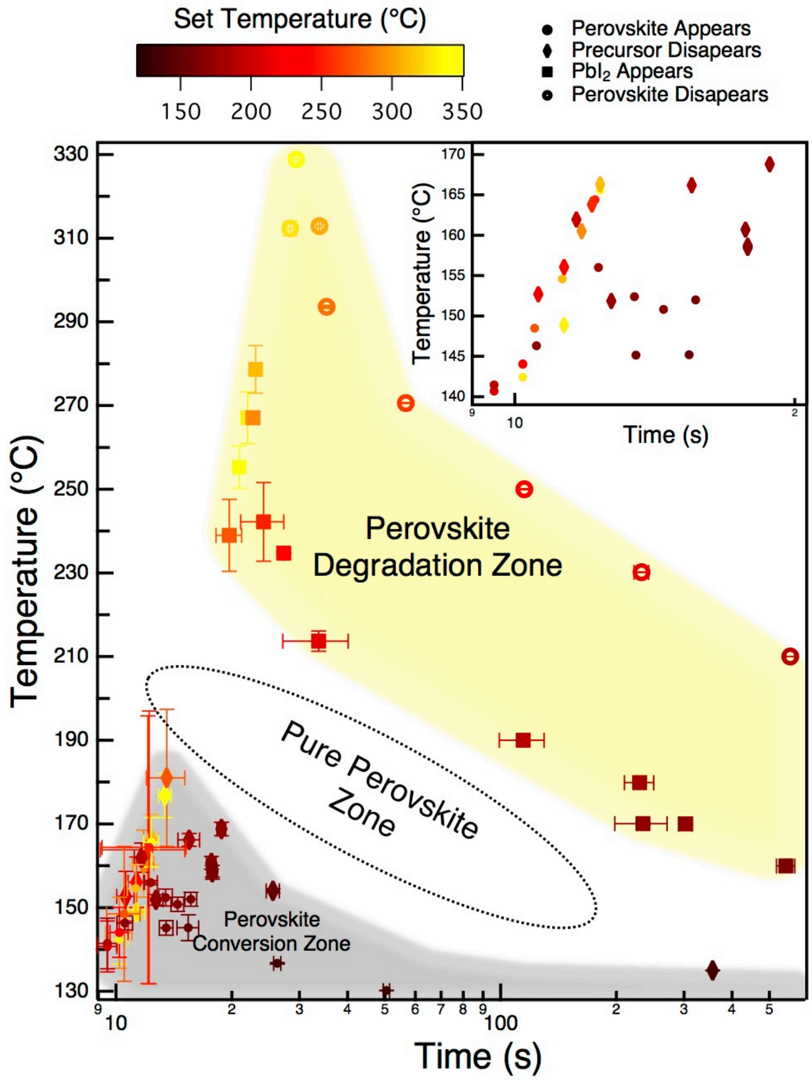

Supplementary Figure 6 | Conversion Zone Diagram: Measured time and temperature, and corresponding perovskite phase conversion stage. The zone highlighted in gray is where the phase transition from the precursor to perovskite takes place. The zone highlighted in yellow is where the degradation from perovskite to PbI_2 takes place and between these two regions is where the samples are in the pure perovskite phase. Solid circles represent where the perovskite phase first appears, the diamonds where the precursor disappears, the squares where the PbI_2 first appears, and the circles where PbI_2 disappears. The color of the marker indicates the set temperature for a given data set. The zones of phase stability are interpolated from these indicators. The insect shows the conversion temperatures within first 20 s of the annealing.

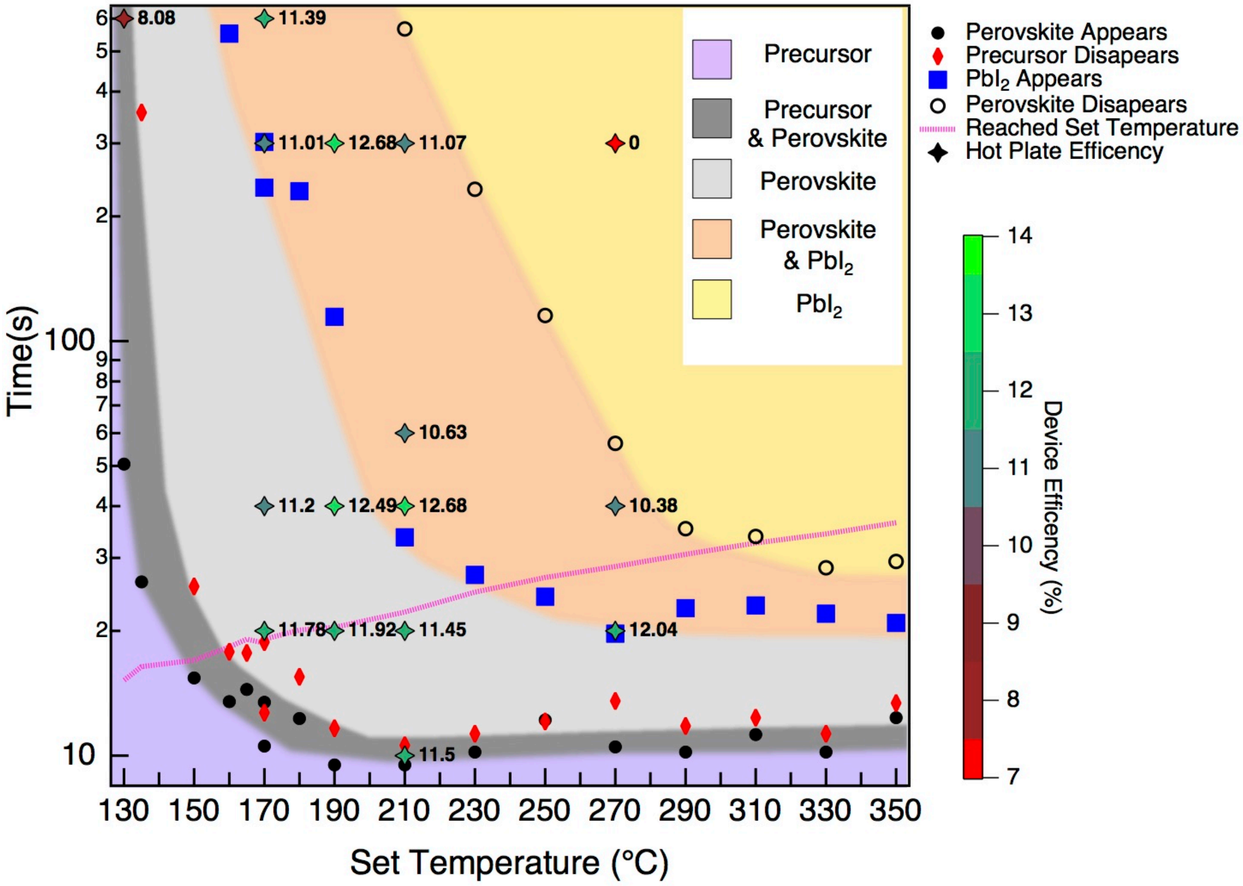

Supplementary Figure 7 | Processing Phase Space and hotplate annealed device performance. The various phases are obtained by interpolating the appearance and disappearance of the hexagonal precursor, perovskite and PbI_2. Red diamonds show precursor disappearance. Solid black circles and open circles denote perovskite appearance and disappearance, respectively. PbI_2 appearance is shown by blue squares. The time when the samples reach the set point temperature is shown by the pink line. The stars show the power conversion efficiency, obtained from *J*-*V* reversely scanned.

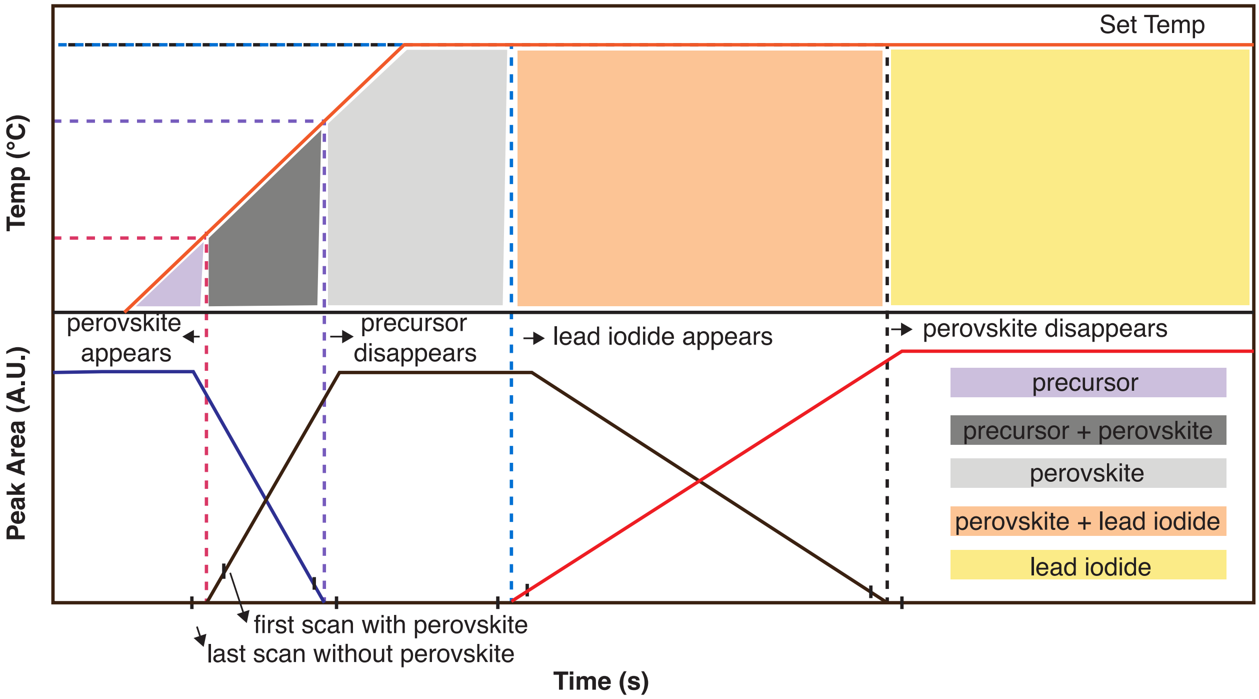

Supplementary Figure 8 | Phase Time and Temperature Determination Schematic. The figure indicates how the time and temperature was determined for appearance/ disappearance of the various phases. For the specific case of the perovskite disappearance, the time is midway between the first scan where the perovskite is observed and the last scan where it is not apparent.
